# Clinical and diagnostic aspects of feline cutaneous leishmaniosis in Venezuela

**DOI:** 10.1186/s13071-018-2747-2

**Published:** 2018-03-20

**Authors:** Aruanai Kalú Rivas, Magdalena Alcover, Pamela Martínez-Orellana, Sara Montserrat-Sangrà, Yaarit Nachum-Biala, Mar Bardagí, Roser Fisa, Cristina Riera, Gad Baneth, Laia Solano-Gallego

**Affiliations:** 1 Department of Animal Medicine and Surgery, Veterinary School, University Centroccidental Lisandro Alvarado, Barquisimeto, Venezuela; 2 Departament de Medicina i Cirurgia Animals, Facultat de Veterinària, Universitat Autònoma de Barcelona, Bellaterra, Spain; 3 Laboratori de Parasitologia, Departament de Biologia, Salut i Medi Ambient, Facultat de Farmàcia i Ciències de l’Alimentació, Universitat de Barcelona, Barcelona, Spain; 4 Koret School of Veterinary Medicine, The Hebrew University, Rehovot, Israel

**Keywords:** *Leishmania mexicana*, *Leishmania infantum*, *Leishmania braziliensis*, Cats, Nodular-ulcerative lesions, ELISA, qPCR, Immunohistochemistry, Venezuela

## Abstract

**Background:**

Venezuela is an endemic area for human and canine leishmaniosis due to *Leishmania infantum* and parasites of the *Leishmania braziliensis* and *L. mexicana* complexes. Limited data are available on feline leishmaniosis (FeL) in this region. The aim of this study was to describe clinical and diagnostic aspects of FeL in Venezuela.

**Results:**

Thirty-one domestic cats from urban areas of Lara State in Venezuela were enrolled. Twenty-five were healthy. Six other cats had solitary or multiple nodular lesions, which were located on the nose, ears, tail and lower limbs. Cutaneous lesions were characterized by diffuse pyogranulomatous infiltrate in all sick cats with numerous intracellular and extracellular amastigotes, and immunohistochemistry was positive for *Leishmania* in five sick cats. All healthy cats were seronegative for *L. infantum* and *L. braziliensis* antigens by ELISA. Two out of five sick cats yielded a positive ELISA result to both *Leishmania* antigens with higher antibody levels to *L. braziliensis* compared to *L. infantum*. Significantly higher antibody levels by ELISA as well as a higher number of bands by Western blot (WB) were found for *L. braziliensis* when compared to *L. infantum* antigens in all sera from Venezuelan sick and healthy cats. All healthy cats were blood *Leishmania* spp*.* qPCR negative while three out of six sick cats were blood qPCR positive. All paraffin-embedded skin biopsies (*n* = 4) as well as cutaneous cytology (*n* = 3) were positive by *Leishmania* spp. qPCR in sick cats. *Leishmania* speciation was obtained only from the cutaneous lesion samples from cytological preparations of two out of three sick cats which were identified as infected with *L. mexicana* or a closely related specie.

**Conclusions:**

Feline leishmaniosis should be included in the differential diagnosis list of nodular-ulcerative lesions. The most reliable diagnostic technique in sick cats is cytological or histopathological examination along with immunohistochemistry, since blood PCR and serology by ELISA might be negative. WB appears to be more sensitive in detecting infection. Cats with leishmaniosis from Venezuela are most likely infected with species of *L. mexicana* or a closely related species or the *L. braziliensis* species complex and not with *L. infantum*.

## Background

Leishmaniosis is a parasitic disease caused by an obligate intracellular protozoan of the genus *Leishmania* (Kinetoplastida: Trypanosomatidae) [1]. In humans, it is manifested clinically in multiple forms including the cutaneous, mucosal and visceral leishmaniosis [2]. The cutaneous form of leishmaniosis in the Eastern Hemisphere is caused by *Leishmania tropica*, *Leishmania major* and *Leishmania aethiopica*, as well as *Leishmania infantum* and *Leishmania donovani*. The *Leishmania* species found in America (the Western Hemisphere) are either in the subgenus *Leishmania* represented by the *L. mexicana* species complex (*L. mexicana*, *L. amazonensis*, *L. pifanoi*, *L. garnhami*, *L. aristidesi* and *L. venezuelensis*) or in the subgenus *Viannia* represented by the *L. braziliensis* species complex [*L.* (*V.*) *braziliensis*, *L.* (*V.*) *guyanensis*, *L.* (*V.*) *panamensis*, and *L.* (*V.*) *peruviana*)] [3].

Cutaneous lesions are the most common clinical sign of leishmaniosis both in human [4] and canine patients [5] for certain *Leishmania* species such as *L. infantum* and the *L. mexicana* and *L. braziliensis* species complexes. Over 200,000 people develop dermal and mucosal leishmaniosis annually in Central and South America [6, 7]. Venezuela is an endemic area of American human cutaneous leishmaniosis (ACL). A wide distribution of ACL has been observed, but the localized clinical form tends to concentrate in states with mountainous geography especially in the Andes (Trujillo, Mérida, Lara and Táchira). It is interesting to note that Lara and Mérida states are characterized by a fairly stable endemic situation that represents almost a third of all national cases [8]. Additionally, cutaneous lesions have been reported in association with *L. venezuelensis*, in the endemic focus of ACL both in humans and domestic animals such as cats in Barquisimeto, Lara State, Venezuela [9]. Moreover, description of human cutaneous leishmaniosis caused by *L. infantum* is also documented [10]. However, *Viannia* is the most relevant subgenus in this territory and is also responsible for metastatic human mucosal leishmaniosis, the severe form of tegumentary disease [11, 12].

Feline leishmaniosis has been described with both visceral and cutaneous forms by demonstration of the presence of the parasite in cats. Several cases in domestic cats have been globally reported, also in America and especially in endemic areas of Central America [13], South America such as Brazil [14, 15] and Paraguay [16] and also in the Mediterranean basin [17] and the Middle East [18]. Nonetheless, the real susceptibility of cats to infection by *Leishmania* spp., their role as reservoir hosts, and the outcome of leishmaniosis in these animals, are poorly understood [19]. Several *Leishmania* species such as *L. mexicana* [20], *L. venezuelensis* [21], *L. braziliensis* [22–24], *L. amazonensis* [25] and *L. infantum* [15, 26–29] have been identified to infect cats in Central and South America.

The most frequently described lesions in FeL are ulcerocrusting, nodular dermatitis, alopecia and scaling [30–32] while the visceral form of the disease involving the spleen, liver, lymph nodes, bone marrow, eye and kidney is less commonly diagnosed [33, 34]. Although clinical cases of leishmaniosis have been reported in cats with coinfection of feline leukemia virus (FeLV) and feline immunodeficiency virus (FIV), the true association between FeL and retroviral infections remains unclear [32, 35].

The laboratory tests recommended for diagnosis of FeL due to *L. infantum* include demonstration of the presence of the parasite by direct microscopic examination in stained smears, and/or culture, serological tests [indirect fluorescent antibody test (IFAT), enzyme-linked immunosorbent assay (ELISA) and Western blot (WB)], and molecular techniques such as the polymerase chain reaction (PCR) [36]. However, limited information is available regarding the diagnosis of other species of *Leishmania* such as *L. braziliensis* infection in dogs and cats [37].

Only very limited data are available on FeL in Venezuela [9]. The identification of clinical manifestations associated with *Leishmania* spp*.* infection in domestic cats in endemic areas as well as the best diagnostic techniques to be employed are crucial topics. Therefore, the aim of this study was to describe clinical and diagnostic aspects of FeL in an endemic area of American cutaneous leishmaniosis, the state of Lara in Venezuela.

## Methods

### Cats and sampling

Thirty-one outdoor domestic short hair cats from urban areas (Barquisimeto, Cabudare, Quibor) of Venezuela Lara State were enrolled. A full physical examination was performed, and breed, age and gender were recorded before sampling. Twenty-five cats were apparently healthy, and 6 cats presented cutaneous lesions. Blood samples (*n* = 31) were collected into ethylenediaminetetraacetic acid (EDTA) for DNA extraction and quantitative PCR (qPCR) and into plain tubes to obtain sera samples from 30 cats for Western blot (WB) and enzyme-linked immunosorbent assay (ELISA) and stored at -20 °C before use. Unfortunately, hematological and biochemical profiles were not performed.

### Diagnosis of *Leishmania* infection based on cytology, histopathology and immunohistochemistry of cutaneous lesions

Impression smears and fine needle aspirates from ulcerative nodular lesions from 6 sick cats were sampled and stained with a Romanowsky stain variant (Diff-Quick). Macroscopical skin lesions of solitary or multiple nodular and ulcerative areas were biopsied in 5 cats. Skin biopsies were fixed in 10% neutral buffered formalin. The dermal inflammatory pattern and the cell populations were evaluated histologically in hematoxylin and eosin (HE)-stained sections. A deparaffinization step was performed on the paraffin blocks of skin biopsies before *Leishmania* immunohistochemistry (IHC). Later, a standard staining protocol with AutostainerPlus (Dako Denmark A/S, Glostrup, Denmark) using rabbit polyclonal antibodies to *L. infantum* was followed. Sections were then counterstained with hematoxylin and cover-slipped for their interpretation [38].

### Detection of antibodies against *L. infantum* and *L. braziliensis* antigens by quantitative enzyme-linked immunosorbent assay (ELISA)

A *Leishmania infantum* in-house ELISA protocol previously described for cats [17] was slightly modified and *L. infantum* (MHOM/MON-1/LEM 75) and *L. braziliensis* (MHOM/BR/88/BCN-25) antigens were used in the same ELISA plate. Cat sera were diluted to 1:800 in phosphate-buffered saline (PBS) with 0.05% Tween 20 (Sigma-Aldrich, St. Louis, Missouri, USA) containing 1% of dry milk and incubated in sonicated crude *L. infantum* and *L. braziliensis* antigen-coated each in half plates (20 μg/ml) for 1 h at 37 °C.

All plates included serum from a sick cat from Cyprus with a confirmed infection with *L. infantum* as a positive control [39] and serum from a healthy cat as a negative control. All samples were analysed in duplicate. The result was quantified as ELISA units (EU) related to a positive feline serum used as a calibrator and arbitrarily set at 100 EU [40] for both antigens.

The cut-off for *L. infantum* was established at 9.2 EU (mean + 4 SD of values from 80 cats from the UK, a non-endemic area). Sera were classified as being positive, when having a value equal or higher than 15.3 EU and negative with 9.2 EU. Values in between were considered doubtful.

The cut-off for *L. braziliensis* was established at 13.8 EU (mean + 4 SD of values from 80 cats from the UK, a non-endemic area). Sera were classified as positive when having a value equal or higher than 21.0 EU and negative with 13.8 EU. Values in between were considered doubtful.

### Western blot (WB)

Sera from 25 apparently healthy cats and five cats with lesions compatible with cutaneous leishmaniosis from Lara State in Venezuela were assessed by WB. In addition, sera from 8 cats from the Queen Mother Hospital at the Royal Veterinary College (RVC), University of London, were used as negative controls from cats living in a non-endemic area of leishmaniosis. Sera from 8 cats from Catalonia in Spain, of which 6 cats were seropositive to *L. infantum* by ELISA, one presenting a doubtful result and one being negative, were also evaluated. Cats from Catalonia (a non-endemic area for *L. braziliensis* infection) were included to compare the pattern of WB with cats from Venezuela. The seropositive cats to *L. infantum* from Catalonia were diagnosed with clinical leishmaniosis and used as positive controls for *L. infantum* antigen.

Western blot was performed with *L. infantum* (MHOM/MON-1/LEM 75) and *L. braziliensis* (MHOM/BR/88/BCN-25) promastigotes as antigens [41, 42]. Sera from cats with leishmaniosis that reacted with polypeptides of low molecular mass (< 36 kDa) of *L. braziliensis* or *L. infantum* antigens were considered positive for WB due to the fact that these antigens are the most specific fractions in diagnosis of FeL [17, 43].

### DNA extraction from blood, paraffin-embedded skin biopsies and cytology from skin lesions

#### Blood DNA purification

DNA was extracted using the Gen Elute blood genomic DNA kit (Sigma-Aldrich) from 31 blood samples. Blood from a non-infected clinically healthy cat was included as negative control every time that DNA extraction was performed [44].

#### Purification of genomic DNA from formalin-fixed, paraffin-embedded skin biopsies

A deparaffinization step was performed on the paraffin blocks of skin biopsies from 4 sick cats (FeV2, FeV3, FeV5 and FeV6) using buffer (20 mM Tris-HCL Ph 8.5; 1 mM EDTA), heating for 10 min at 95 °C and centrifuging for 20 min at 12,000×*g*. Then, DNA extraction was performed using the QIAamp® DNA FFPE (Qiagen, Hilden, Germany) in accordance with the manufacturer’s recommendations.

#### Purification of genomic DNA from cytological slides from skin lesions

DNA extraction was performed from cutaneous lesions from cytological slides from 3 sick cats (Fev3, Fev4 and Fev5) with QIAamp® DNA Mini and Blood (Qiagen) following the manufacturer’s instructions. A scalpel (Braun, Tuttlingen, Germany) was used to obtain tissue in a tube from each sample. Twenty microliters of protease, 200 μl of PBS and 200 μl of lysis buffer (Buffer AL) were added and vortexed. Samples were incubated at 56 °C.

### *Leishmania* spp*.* kinetoplast quantitative polymerase chain reaction (qPCR)

The presence of *Leishmania* spp. DNA in blood samples (*n* = 31), paraffin embedded skin biopsies (*n* = 4) and cytological slides from cutaneous lesions (*n* = 3) was initially analysed by amplification of kinetoplast DNA sequence by a real-time polymerase chain reaction (qPCR). Each amplification was performed in triplicate, in 20 μl reaction, 15 pmol of direct primer (5'-CTT TTC TGG TCC TCC GGG TAG G-3'), 15 pmol of reverse primer (5'-CCA CCC GGC CCT ATT TTA CAC CAA-3'), 50 pmol of the labelled TaqMan probe (FAM-TTT TCG CAG AAC GCC CCT ACC CGC-TAMRA) and 5 μl of sample DNA. Amplification and detection were performed in the ABI Prism 7700 system (Applied Biosystems, Foster City, CA, USA.) in two-step temperature (94 °C and 55 °C) cycling over 45 cycles. Positive controls (DNA from *L. infantum* MHOM /ES /04 /BCN-61) and negative controls were included in each RT-PCR analysis [45].

### Internal transcribed spacer 1 (ITS1) restriction fragment length polymorphism (RFLP), quantitative PCR (qPCR), sequencing and phylogenetic analysis

The species identification of the *Leishmania* isolates was performed on DNA from cutaneous lesions (cytological preparations) from 3 sick cats from Venezuela (FeV3, FeV4 and FeV5) and on DNA from cutaneous lesions (skin paraffin-embedded biopsies) from 4 sick cats (FeV2, FeV3, FeV5 and FeV6). Two different techniques were performed.

#### Polymerase chain reaction-restriction fragment length polymorphism (PCR-RFLP) analysis of amplified ITS-1 sequences

For the identification of *Leishmania* species, we amplified the ribosomal ITS-1 region with primers LITSR (5'-CTG GAT CAT TTT CCG ATG-3') and L5.8S (5'-TGA TAC CAC TTA TCG CAC TT-3') [46]. Amplification reactions were performed in volumes of 50 μl containing 3 μl of isolated DNA, 5 μl of 10× buffer (BIOTAQ DNA Polymerase, Bioline, London, UK), 1.5 mM MgCl_2_, 0.2 mM dNTP, 0.2 mM of each primer and 1.5 units of Taq polymerase (BIOTAQ DNA Polymerase, Bioline). A denaturing step at 95 °C for 2 min, followed by 35 cycles of denaturing for 20 s at 95 °C, annealing for 30 s at 53 °C, and extension for 1 min at 72 °C, followed by a final extension at 72 °C for 1 h was carried out in thermal cycler (MJ Research PTC-200 DNA Engine, Alameda, CA, USA). DNA samples extracted from promastigote cell cultures of *L. infantum*, *L. tropica*, *L. major* and *L. braziliensis* were used as positive controls. A non-template control with the same reagents described above but without DNA was added to PCR to rule out contamination.

The PCR products, previously digested with the restriction enzyme *BsuR*I (*Hae*III), were separated by electrophoresis in 2% wide-range agarose (Sigma) at 150 V in SGTB 1× buffer (GRISP LDA, Research Solutions, Porto, Portugal). A solution of SYBR safe DNA gel stain (Invitrogen Ltd., Paisley, UK) was used to visualize the separated DNA fragments under UV light [47].

#### Quantitative PCR high-resolution melting (qPCR-HRM) *Leishmania* genotyping based on a ITS1, sequencing and phylogenetic analysis

A fragment of ITS1 region of the leishmanial ribosomal RNA operon was amplified (265–288 bp) by real-time PCR using primers ITS-219F (5'-AGC TGG ATC ATT TTC CGA TG-3') and ITS-219R (5'-ATC GCG ACA CGT TAT GTG AG-3') and then evaluated by high resolution melting (HRM) analysis as previously reported [48]. DNA samples extracted from promastigote cell cultures of *L. infantum*, *L. tropica* and *L. major* were used as positive controls for each corresponding PCR reaction and DNA from colony-bred dogs negative by PCR for vector-borne pathogens was used as a negative control. A non-template control (NTC) with the same reagents described above but without DNA was added to each PCR to rule out contamination.

All positive PCR products were sequenced using the BigDye Terminator v.3.1 Cycle Sequencing Kit and an ABI PRISM 3100 Genetic Analyzer (Applied Biosystems), at the Center for Genomic Technologies, Hebrew University of Jerusalem, Israel. DNA sequences were evaluated with the ChromasPro software version 2.1.1 (Technelysium Pty Ltd., South Brisbane, Australia) and compared for similarity with sequences available on the GenBank, using BLAST program (https://blast.ncbi.nlm.nih.gov/Blast.cgi).

Phylogenetic analysis was performed by MEGA6 [49] using the Maximum Likelihood method based on the Tamura 3-parameter model [50]. Initial phylogenetic trees for the heuristic search were obtained by applying the Neighbor-Joining method to a matrix of pairwise distances estimated using the Maximum Composite Likelihood (MCL) approach. The bootstrap consensus tree inferred from 1000 replicates was taken to represent the evolutionary history of the taxa analyzed [51] and branches corresponding to partitions reproduced in less than 60% bootstrap replicates were collapsed.

### Detection of FeLV antigen and FIV antibody

In order to evaluate retroviral infections to rule out concomitant infections the same 30 cats from Venezuela described above (5 sick cats and 25 apparently healthy cats) were tested serologically for FeLV antigen and FIV antibody. Detection of FeLV p27 antigen and anti-FIV antibodies was performed by a commercial ELISA (INGEZIM FeLV and INGEZIM FIV®, Ingenasa, Madrid, Spain) according to the manufacturer’s protocol.

### Statistical analysis

Statistical analysis was performed using the SPSS 17.0 software for Windows (SPSS Inc., Chicago, USA). A non-parametric Mann-Whitney U-test was used to compare groups. A non-parametric Wilcoxon signed-rank test was used to compare paired continuous variables. Differences were considered significant with a 5% significance level (*P* < 0.05. The descriptive statistical analysis was conducted with R project software (2017).

## Results

### Cats

**Fig. 1:**
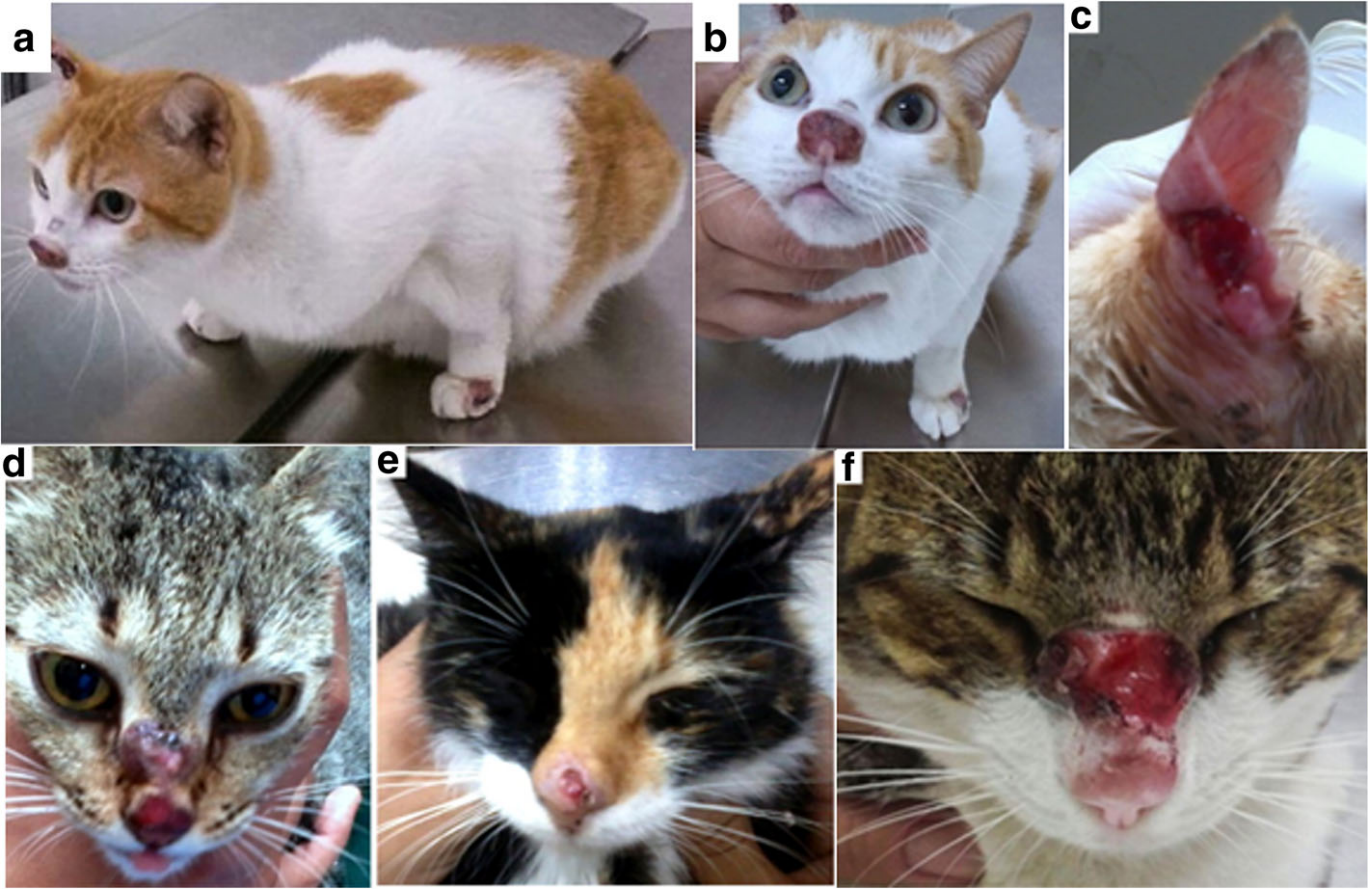
Cats with cutaneous leishmaniosis: **a** Adult female cat with ulcerative nodular lesions in the nose, front leg and pinna (ID: FeV2). **b** Close up of the same cat (ID: FeV2). **c** Adult female cat, ulcerative lesion in the pinna (ID: FeV4). **d** Adult female cat with a nasal ulcer (ID: FeV6). **e** Adult female cat with an ulcerative nodular lesion on the nose (ID: FeV5); **f** 8-month-old male cat with an ulcerated nodule on the nose (ID: FeV3)

Physical examination of all cats included in this study determined that 24 were adults, 5 were old cats and 2 were young cats. Moreover, the distribution according to gender was 13 females and 18 males. The majority of cats did not present any systemic clinical sign or dermatological lesions compatible with leishmaniosis and were classified as apparently healthy (25/31, 80.6%). All apparently healthy cats were owned cats from Barquisimeto and Cabudare cities from Lara State. They were 9 females (*n* = 2, old cats; *n* = 7, adults) and 16 males (*n* = 3, old cats; *n* = 12, adults; and *n* = 1 young cat). On the other hand, 6 of the 31 cats (6/31, 19.3%) presented dermatological clinical signs. Signalment, geographical location and clinical description are summarized in Table 1. Sick cats were all stray cats living in cat colonies. They were 4 females and 2 males all adults, except for one 8-month-old young male. Skin lesions consisted of solitary or multiple nodular lesions (Fig. 1) which were located on the nose (*n* = 3) (Fig. 1d-f); ears (*n* = 1) (Fig. 1c); ears and nose (*n* = 1) (Fig. 1a, b); and nose, ears, tail and lower limbs (*n* = 1). Cats did not show other clinical signs. Those cats did not receive any treatment and were humanely euthanized.

**Table 1:** Summary of signalment, clinical findings and diagnostics tests results in six cats with cutaneous leishmaniosis

Cat ID	Signalment and clinical description (city)	ELISA (EU)		Western blot (bands kDa)		Microscopical observations			*Leishmania* spp*.* kinetoplast qPCR			*Leishmania* spp. ITS1 qPCR	
		*L. infantum*	*L. braziliensis*	*L. infantum*	*L. braziliensis*	Cytology	Biopsy H/E	IHC	Blood	Skin lesions		Skin lesions	
										Biopsy	Cytology	Biopsy	Cytology
FeV1	Adult, male, ulcerative nodular lesion on the nose (Cabudare)	− 4.5	− 7.1	–	+ (14, 18, 22, 38)	–	–	+	–	np	np	np	np
FeV2	Adult, female, ulcerative nodular lesion in the nose pinna and interdigital area (Cabudare)	− 2.4	− 3.7	–	–	–	+	+	–	+	np	–	np
FeV3	8 months male ulcerated nodule on the nose (Quíbor)	+ 22.5	+ 27.2	+ (16–20, 24–30, 46)	+ (14–20, 24–36, 42, 46–52)	+	+	+	–	+	+	–	+^b^
FeV4^a^	Adult, female, ulcerative lesion in the pinna (Quíbor)	np	np	np	np	+	np	np	+	np	+	np	+^c^
FeV5	Adult, female, ulcerative nodular lesion in the nose (Barquisimeto)	− 6.2	− 9.0	–	+ (18, 24, 28, 65, 70)	+	+	+	+	+	+	–	–
FeV6	Adult, female nasal ulcer (Barquisimeto)	+ 38.0	+ 52.5	–	+ (16, 28, 30–36, 42, 46, 70)	+	+	+	+	+	np	–	np

*Abbreviations*: *np* not performed, + positive, − negative

^a^Serum was not available

^b^100% identity with *L. mexicana* ITS1 (AB558250.1) identified also as *L. mexicana* by RFLP

^c^93% identity with *L. mexicana* ITS1 (GenBank: AB558250.1)

### Cytology, histopathology and immunohistochemistry

**Fig. 2:**
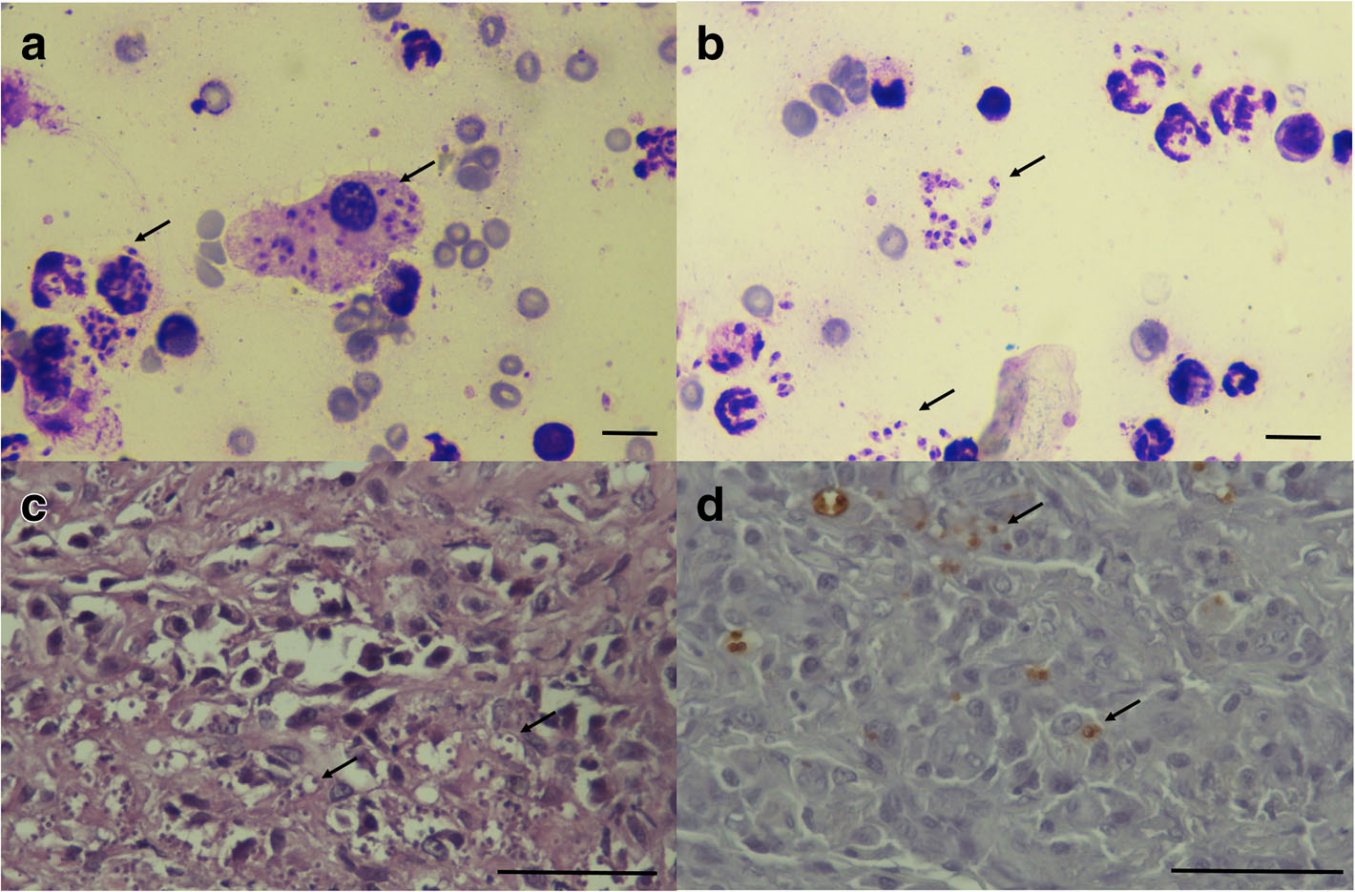
**a**, **b** Cytology from cutaneous lesions from cat ID Fev3 with macrophagic-neutrophilic inflammation, showing numerous intracellular and extracellular *Leishmania* amastigotes (arrows) (diff quick stain 1000×). *Scale-bars*: 10 μm. **c** Diffuse pyogranulomatous inflammatory infiltrate from cat ID Fev5 with numerous *Leishmania* amastigotes (arrows) (H&E 400×). **d** Positive immunohistochemistry for *Leishmania* amastigotes from cat ID Fev5 (brown dots are indicated with arrows) (400×). *Scale-bars*: 50 μm

Cytology was performed from cutaneous lesions in 4 sick cats. In the majority of cases, mixed inflammation with predominance of macrophages and neutrophils was found. Numerous intracellullar and extracellular *Leishmania* amastigotes were also noted (Fig. 2a, b). Histologically, cutaneous lesions from 4 sick cats were characterized by epidermal hyperplasia and hyperkeratosis. Diffuse infiltrate with predominance of macrophages and plasma cells with numerous intracellular and extracellular amastigotes were observed (Fig. 2c). Occasionally, mast cells, lymphocytes and eosinophils were also encountered. Additionally, one sick cat presented crusting and necrosis, and presence of amastigotes was not observed. Immunohistochemistry was positive for *Leishmania* spp. in the 5 biopsied cats (Fig. 2d).

### ELISA

All apparently healthy cats (*n* = 25) were negative by ELISA for *L. braziliensis* (mean ± SD = 4.5 ± 2.6 EU) and most of them (*n* = 22) were negative for *L. infantum* (mean ± SD = 2.5 ± 1.5 EU) antigens. Interestingly, when all sera samples (*n* = 30) were analysed, statistically significant higher antibody levels were found for *L. braziliensis* (mean ± SD = 7.0 ± 9.8 EU) when compared to *L. infantum* (mean ± SD = 4.5 ± 7.6 EU) antigen (Wilcoxon signed-rank test: *Z* = -4.679, *P* < 0.0001). Additionally, 3 out of 25 apparently healthy cats and one sick cat presented doubtful results for *L. infantum* antigen. There were no animals with doubtful results for *L. braziliensis* antigen*.* Specific antibody response was significantly higher in sick cats when compared to healthy cats to both *L. braziliensis* (Mann-Whitney U-test: *Z* = -2.47, *P* = 0.01) and *L. infantum* (Mann-Whitney U-test: *Z* = -2.69, *P* = 0.05) antigens. Two out of 5 sick cats yielded positive ELISA result to both *Leishmania* antigens (*L. infantum*: mean ± SD = 30.2 ± 10.9 EU and *L. braziliensis*: mean ± SD = 39.9 ± 17.9 EU) while the rest were seronegative (Table 1). Also, higher antibody levels were found for *L. braziliensis* (mean ± SD = 19.9 ± 20.3 EU) antigen than to *L. infantum* (mean ± SD = 14.7 ± 15.2 EU) antigen when all sick cats were evaluated for ELISA (Wilcoxon signed-rank test: *Z* = -2.023, *P* = 0.043)*.*

The cats that were seropositive to *L. infantum* antigen from Catalonia were diagnosed with clinical leishmaniosis. When those animals were tested serologically with *L. braziliensis* antigen, 5 out of 8 presented negative ELISA results, one had a doubtful result and two showed positive results.

### Western blot

As expected, sera from cats from the UK did not react with any polypeptides from both antigens. Bands recognized for *L. braziliensis* and *L. infantum* antigens by cat sera from Venezuela and Catalonia are described in Table 2. In the case of the Venezuelan samples, the highest sensitivity for *L. braziliensis* antigen was found in the following fractions: 70, 65, 52, 50, 46, 42, 36, 34, 30, 28, 18 and 16 kDa. The highest sensitivity for *L. infantum* antigen in Venezuelan cats was found in the following fractions: 70, 65, 46, 34, 30, 28, 24, 18 and 16 kDa (Table 2). In contrast, Catalonian samples recognized a higher number of bands for *L. infantum* antigen (70, 65, 52, 46, 28, 24, 20, 18, 16 and 14 kDa) when compared to *L. braziliensis* antigen (70, 68, 65, 16 and 14 kDa).

**Table 2:** Antibody recognition of *L. infantum* and *L. braziliensis* antigens by WB in sera of cats from Venezuela and Catalonia (Spain)

WB band (kDa)	*Leishmania braziliensis* antigen						*Leishmania infantum* antigen					
	Total no. of cats (*n* = 38)		Endemic area Catalonia (*n* = 8)		Endemic area Venezuela (*n *= 30)		Total no. of cats (*n *= 38)		Endemic area Catalonia (*n *= 8)		Endemic area Venezuela (*n *= 30)	
	*n*	%	*n*	%	*n*	%	*n*	%	*n*	%	*n*	%
70	11	12	2	10	9	13	5	9	1	4	4	11
68	2	2	2	10	2	3	0	0	0	0	0	0
65	7	8	2	10	5	7	3	5	1	4	3	8
58	1	1	0	0	1	1	0	0	0	0	0	0
56	2	2	0	0	2	3	0	0	0	0	0	0
52	5	5	0	0	5	7	1	2	1	4	0	0
50	3	3	0	0	3	4	0	0	0	0	0	0
48	2	2	0	0	2	3	0	0	0	0	0	0
46	6	7	1	5	5	7	3	5	1	4	2	6
44	2	2	2	10	0	0	1	2	0	0	1	3
42	2	2	0	0	2	3	0	0	0	0	0	0
40	1	1	1	5	0	0	0	0	0	0	0	0
38	1	1	0	0	1	1	0	0	0	0	0	0
36	3	3	1	5	2	3	0	0	0	0	0	0
34	4	4	1	5	3	4	7	12	3	13	4	11
30	6	7	1	5	5	7	5	9	3	13	2	6
28	7	8	1	5	6	8	6	10	1	4	5	14
24	3	3	1	5	2	3	5	9	2	9	3	8
22	1	1	0	0	1	1	0	0	0	0	0	0
20	3	3	0	0	3	4	3	5	2	9	1	3
18	6	7	1	5	5	7	5	9	2	9	3	8
16	8	9	2	10	4	6	11	19	4	17	7	19
14	5	5	2	10	3	4	3	5	2	9	1	3
Total no. of bands	91		20		71		58		23		36	
Mean no. of bands	3.9		0.8		3.0		2.5		1.5		1	

*Note*: Statistical results: *L. braziliensis* bands: Venezuelan cats > Catalonian cats (Mann-Whitney U-test: *Z* = -4.03, *P* = 0.0001). Venezuelan cats: *L. braziliensis* > *L. infantum* (Wilcoxon signed-rank test: *Z* = -3.15, *P* = 0.02)

The majority of sick cat sera from Venezuela recognized variable patterns of polypeptides with molecular masses ranging between 14–70 kDa for *L. braziliensis* antigen while they recognized polypeptide from *L. infantum* antigen less frequently (Table 1).

There was a statistically significant predominance of bands specific for *L. braziliensis* antigen in cats from Venezuela when compared to Catalonian cats (Table 2) (Mann-Whitney U-test: *Z* = -4.03, *P* = 0.0001). Also, when sick Venezuelan cats were compared to seropositive cats from Catalonia, a high number of bands for *L. braziliensis* antigen was observed (Mann-Whitney U-test: *Z* = -2.55, *P* = 0.01) (Table 3).

**Table 3:** Antibody recognition of *L. braziliensis* and *L. infantum* antigens by WB in seropositive and sick cats from Venezuela and Catalonia (Spain)

WB band (kDa)	*Leishmania braziliensis* antigen						*Leishmania infantum* antigen					
	Total no. of cats (*n* = 10)		Endemic area Catalonia (*n *= 5)		Endemic area Venezuela (*n *= 5)		Total no. of cats (*n *= 10)		Endemic area Catalonia (*n *= 5)		Endemic area Venezuela (*n *= 5)	
	*n*	%	*n*	%	*n*	%	*n*	%	*n*	%	*n*	%
70	5	9	2	10	3	8	3	8	1	0	2	13
68	3	5	2	10	1	3	0	0	0	0	0	0
65	4	7	2	10	2	6	3	8	1	0	2	13
58	1	2	0	0	1	3	0	0	0	0	0	0
56	1	2	0	0	1	3	0	0	0	0	0	0
52	1	2	0	0	1	3	1	3	1	0	0	0
50	1	2	0	0	1	3	0	0	0	0	0	0
48	1	2	0	0	1	3	0	0	0	0	0	0
46	3	5	1	5	2	6	3	8	1	0	2	13
44	2	4	2	10	0	0	1	3	0	0	1	7
42	2	4	0	0	2	6	0	0	0	0	0	0
40	1	2	1	5	0	0	0	0	0	0	0	0
38	1	2	0	0	1	3	0	0	0	0	0	0
36	3	5	1	5	2	6	0	0	0	0	0	0
34	3	5	1	5	2	6	3	8	3	1	0	0
30	3	5	1	5	2	6	4	11	3	1	1	7
28	4	7	1	5	3	8	4	11	1	0	3	20
24	3	5	1	5	2	6	3	8	2	0	1	7
22	1	2	0	0	1	3	0	0	0	0	0	0
20	1	2	0	0	1	3	3	8	2	0	1	7
18	4	7	1	5	3	8	3	8	2	0	1	7
16	4	7	2	10	2	6	5	13	4	1	1	7
14	4	7	2	10	2	6	2	5	2	0	0	0
Total no. of bands	56		20		36		38		23		15	
Mean no. of bands	2		1		2		2		1		1	2

*Note*: Statistical results: *L. braziliensis*-specific bands: Venezuelan sick cats > Catalonian sick cats (Mann-Whitney U-test: *Z* = -2.55, *P* = 0.011). Venezuelan sick cats: *L. braziliensis* > *L. infantum* (Wilcoxon signed-rank test: *Z* = -3.58, *P* =0.0001)

The Venezuelan cats showed a significantly higher number of bands for *L. braziliensis* antigen when compared to *L. infantum* antigen (Wilcoxon signed-rank test: *Z* = -3.15, *P* = 0.02) (Table 2). Additionally, a higher number of bands for *L. brazilienis* antigen were also found when compared with *L. infantum* antigen in Venezuelan sick cats (Wilcoxon signed-rank test: *Z* = -3.58, *P* = 0.0001) (Table 3). No statistical differences were observed within Catalonian cats when *L. braziliensis* and *L. infantum* bands were compared.

Four out of five sick cats from Venezuela resulted positive for *L. braziliensis* [low molecular mass (< 36 kDa)]. In addition, positive results to *L. braziliensis* WB were found in 6 out of 25 (24%) apparently healthy cats from Venezuela and also in 2 sick cats from Catalonia. In the case of cats from Venezuela, 3 sick cats and 7 apparently healthy cats resulted positive for *L. infantum* WB. Five out of eight Catalonian cats presented compatible results for *L. infantum* antigens. In general, the intensity of bands in sick cats increased with an increase in the antibody level.

### Blood and cutaneous lesions *Leishmania* kinetoplast qPCR, ITS1 RFLP and qPCR for identification, sequencing and phylogenetic analysis

All twenty-five clinically healthy cats were blood qPCR negative (25/31 cats, 90.3%). Additionally, when blood from sick cats was analysed, 3 out of 6 cats were *Leishmania* kinetoplast qPCR positive (3/31 cats, 9.7%) while 3 were negative, including 1 seropositive sick cat by ELISA. DNA extraction was performed from paraffin-embedded skin biopsies of 4 sick cats and all samples were positive by kinetoplast qPCR (Table 1). There was no correlation between the pattern of bands recognized by WB and PCR results.

**Fig. 3:**
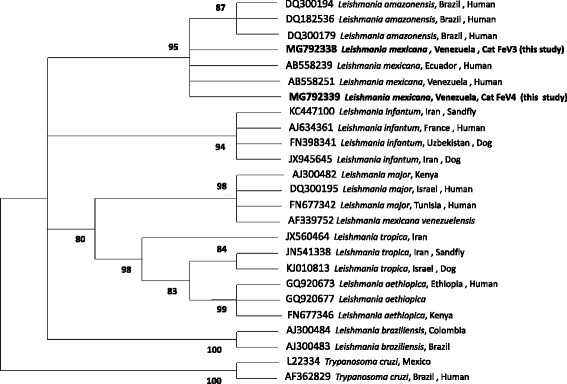
Phylogenetic analysis constructed based on 210 bp DNA sequences of the ITS1 locus of Venezuelan cats (FeV3 and FeV4). Sequences from this study were compared to other sequences deposited in GenBank. Phylogenetic analysis was inferred by using the Maximum Likelihood method based on the Tamura 3-parameter model. The number of bootstrap replicates are 1000 and branches corresponding to less than 60% bootstrap replicates are collapsed. Bootstrap values higher than 60% are indicated next to the branches. GenBank accession number, the strain, country of origin and host from which the sequences were derived are included for each sequence

DNA samples from cytological preparations of cutaneous lesions from 3 cats was extracted and all samples were positive by kinetoplast qPCR (Table 1). Positive DNA samples from cutaneous lesions paraffin-embedded skin biopsies (*n* = 4) and cytological preparations (*n* = 3)] were submitted to parasite species identification by PCR amplifying a fragment of the ITS1 region. Only samples from cytological preparations of 2 cats were positive by ITS1 qPCR-HRM (FeV3 and FeV4) while samples from FeV5 were negative. In addition, the same DNA from the cytological preparation from FeV3 was also confirmed as positive by PCR-RFLP, but FeV4 and FeV5 were PCR-RFLP-negative (Table 1). All DNA samples from paraffin-embedded skin biopsies were negative by ITS1 qPCR-HRM (Table 1) and PCR-RFLP. The DNA sequence of cat FeV3 was 100% identical to a partial *18S* rRNA ITS1 sequence of *L. mexicana* (GenBank: AB558250.1) over 210 bp, as found by BLAST analysis. In contrast, the DNA sequence of cat FeV4 was only 93% identical to a partial *18S* rRNA ITS1 sequence of *L. mexicana* (GenBank: AB558250.1). A phylogenetic tree with the two Venezuelan cats’ results is presented in Fig. 3; in this tree, the DNA sequences from cats FeV3 and FeV4 clustered together with *L. mexicana* sequences from other sources deposited in GenBank.

### Detection of FeLV antigen and FIV antibodies and relationship with *Leishmania* infection

All cats tested (*n* = 30) were negative for FeLV antigenemia. FIV-specific antibodies were found in 2 out of 25 apparently healthy cats tested (6.6%), both of them seronegative for *L. infantum* and *L. braziliensis*-specific antibodies based on ELISA and also negative by kinetoplast qPCR, but when WB positivity was studied, both cats resulted positive to *L. braziliensis*-specific bands and one was positive to *L. infantum-s*pecific bands.

## Discussion

The present study describes a clinical case series of solitary or multiple ulcerative nodular dermatitis due to *Leishmania* spp. in cats from Lara State, Venezuela. Unfortunately, so far, *Leishmania* identification has only been possible from the skin lesion of two cats and identified as *L. mexicana*. To the best knowledge of the authors, we report the first feline case of cutaneous lesions due to *L. mexicana* infection from Lara State, Venezuela. Interestingly, so far cats have only been described to be infected with *L. mexicana* in Texas in the USA [13, 20, 52]. In agreement with our findings, *L. mexicana* infection was previously diagnosed in humans with cutaneous leishmaniosis from Lara State, Venezuela [53] as well as in pools of *Lutzomyia* sand flies from Sucre State in Venezuela [54].

Unfortunately, *Leishmania* identification was not obtained from the remaining sick cats from Venezuela described in the present study. Since formalin-fixation of histological specimens causes partial DNA destruction, which may hamper diagnostic PCR analysis [55, 56], we believe that there was inhibition of DNA PCR-based analyses of histological specimens. Therefore, the amount of DNA amplified was reduced and this did not enable the identification of *Leishmania*. However, based on clinical and serological findings and the geographical distribution of FeL [36], it is likely that species of *L. mexicana* and/or *L. braziliensis* complexes were the cause of infection of the remaining cats described here. As with the cases presented here, clinical disease in cats caused by natural infection with species other than *L. infantum* is typically reported as nodular or ulcerative dermatitis with no systemic clinical signs. Skin lesions are often single but they can metastasize [36].

Solitary cutaneous lesions have been reported in association with *L. venezuelensis* infection, in the endemic focus of ACL both in humans and domestic animals in Barquisimeto, Lara State, Venezuela [9, 53]. Interestingly, in this previous study, four cats were observed with cutaneous nodules on the nose and smaller nodules on the ears, and diffuse nodular lesions on the tail and legs [9]. This clinical presentation was similar with the findings obtained in the present study from cats from the cities of Quíbor, Cabudare and Barquisimeto in Lara State, Venezuela. It is also important to highlight that feline cutaneous lesions described in the present study are similar to the ones described also in humans. It is likely that cats might only be an accidental host of *L. venezuelensis* infection [36]. It is also important to remark that the grouping of *L. mexicana* species complex is still controversial [53]. *Leishmania venezuelensis* was originally described on the basis of distinguishable multilocus enzyme electrophoresis (MLEE) patterns as a species independent of other members of the *L. mexicana* complex [57]. Furthermore, a monoclonal antibody specific for *L. venezuelensis* was developed for identification using immunological methods [58]. However, there are limited molecular data regarding *L. venezuelensis* [59] and some authors strongly suggested that *L. venezuelensis* is a variant of *L. mexicana* [53]*.*

A study carried out in Cojedes State of Venezuela revealed human, dog and equine populations with ulcers, other active lesions, skin scars and mucosal alterations, due to *Leishmania* (*Viannia*) *braziliensis* characterized by zymodeme and serodeme typing [60]. In addition, natural [23, 24] and experimental [19] *L. braziliensis* infections have been described in domestic cats. *Leishmania braziliensis* natural infection in cats has been described in Brazil [60], French Guiana [23] and in northern Argentina [61]. Cutaneous lesions previously described were also similar to the ones reported in the present study. However, the finding of cats with cutaneous leishmaniosis does not reflect an important role of these domestic animals in the natural transmission of the disease in these areas, and these animals probably represent accidental hosts [21].

In the present study, cats presented ulcerative nodular dermatitis mainly on the face. Cutaneous lesions in cats naturally infected with *Leishmania* spp. occur mainly on the nose, followed by the ears or at both sites [2] and also occasionally on the limbs. The skin alterations in FeL are unspecific and can be associated with other clinical conditions [25]. The commonly seen cutaneous nodular form in FeL cases should be distinguished from nodules caused in cats with sterile or eosinophilic granuloma, cryptococcosis, sporotrichosis, histoplasmosis, mycobacterioses, and a wide variety of cutaneous neoplasms, e.g. feline sarcoids, mast cell tumor, fibrosarcoma, basal cell carcinoma, bowenoid *in situ* carcinoma and lymphoma [36]. The main differentials of the ulcerative lesions include squamous cell carcinoma, idiopathic ulcerative dermatitis, herpes virus dermatitis, mosquito-bite dermatitis, atypical mycobacteriosis and feline leprosy, cutaneous vasculitis, erythema multiforme and cold-agglutinin disease. Interestingly, squamous cell carcinoma may co-exist with *Leishmania* infections as clinical case reports due to *L. infantum* have been documented in cats in Europe [62, 63]. In the present study, concomitant disease was not diagnosed in the sick cats studied.

Leishmaniosis is diagnosed by demonstration of the parasite by direct microscopic examination in stained smears, and/or culture of skin lesions, lymph node aspirates, peripheral blood, bone marrow aspirates or indirectly by serological techniques [41]. A clinical form characterized by a very low number of intralesional parasites can be detected by an immunohistochemical technique. This technique is a highly sensitive and specific tool for the diagnosis of canine and feline leishmaniosis [36, 64]. In this study, different techniques were used in the cats with cutaneous lesions, such as cytology, skin biopsy and immunohistochemistry, by which the diagnosis of infection by *Leishmania* spp*.* was made*.* Besides that, serological and molecular diagnostic techniques were also used in sick and apparently healthy cats. One sick cat and 25 clinically healthy cats were negative by serology and blood qPCR. The sick cats did not always give a positive result to blood qPCR and serology. The present findings might indicate that clinically healthy cats are not carriers of *Leishmania* spp. present in Venezuela.

Moreover, the ELISA and qPCR discordant results can be attributed to the inherent differences between serological testing and molecular methods. PCR is a very sensitive technique. However, intermittent parasitemias are very likely in cats as described in dogs [65] and therefore, PCR from blood might not be very sensitive. The parasite load in blood was quite variable in the cats studied and poorly correlated with the degree of antibody levels [17]. The present findings suggest that more than one technique should be used for detection of feline cutaneous leishmaniosis in South America.

*Leishmania* species such as *L. infantum* and *L. braziliensis* among others co-exist in South America and both species can infect cats [36]. However, there are limited studies regarding serological tests that will distinguish between *L. infantum-* and *L. braziliensis-*specific feline antibodies in these regions. Here, we report an ELISA that combines both antigens. Interestingly, higher antibody levels were found using *L. braziliensis* antigen in Venezuelan cats when compared with *L. infantum* antigen. This quantitative in-house ELISA appears to help establishing what *Leishmania* species or closely related *Leishmania* species are most likely infecting cats in the respective endemic areas. These findings are extremely important in areas where several parasite species might co-exist, therefore, this type of ELISA should be used to determine the most likely *Leishmania* parasite infecting dogs and cats in South America. Unfortunately, we did not have antigen of *L. mexicana* to perform WB.

Furthermore, the WB analysis also revealed that Venezuelan cat sera recognized a significantly higher number of *L. braziliensis* polypeptides when compared to *L. infantum* antigen. In addition, the intensity of bands increased with an increase in the antibody level. There was no correlation between the pattern of bands recognized and PCR results. Antigens of low molecular weight (12–14 and 14–18 kDa), seem to be very specific and their recognition in the immunoblot is highly sensitive in the diagnosis of subclinical *Leishmania* infection in dogs and cats [43]. In the cats analysed, similar results were observed. In experimentally infected dogs, antibodies specific for low molecular weight fractions are the first to appear following infection [41]. Based on the results of this study, it appears that the WB enables detection of early phases of infection in apparently healthy cats with negative antibody levels by ELISA or PCR results. It also important to highlight that WB seems to be the best serological technique to be used when testing sick and apparently healthy cats from Venezuela as previously reported for European cats [43]. In agreement with the present study, another study reveals that the use of WB with whole antigen or antigenic Fe-SODe (iron superoxide dismutase) fraction was an optimal method for the detection of FeL [66]. The use of antigenic fractions of cultures of *L. mexicana*, *L. braziliensis* and *L. infantum* showed satisfactory results with high sensitivity, specificity and efficacy for the detection of this disease in cats [66]. Therefore, WB should be widely used in the clinical setting for diagnosis clinical FeL as well as for detection of subclinical infections.

## Conclusions

We conclude that leishmaniosis should be included in the differential diagnosis list of nodular-ulcerative lesions in cats, mainly on the nose and ears. In addition, to the best of our knowledge, we described for the first time, cutaneous lesion associated with *L. mexicana* infection from two Venezuelan cats. The most reliable diagnostic technique in sick cats is cytological or histopathological examination along with immunohistochemistry, since blood PCR and serology by ELISA might be negative. However, WB appears to be more sensitive in detecting infected cats. Based on molecular and serological findings, cats from Venezuela are most likely infected with species of *L. mexicana* or *L. braziliensis* species complexes rather than *L. infantum*. Finally, the present findings might indicate that clinically healthy cats are not carriers of *Leishmania* spp. present in Venezuela.

## Abbreviations

ACL: American human cutaneous leishmaniosis
DAB: diaminobenzidine
ELISA: enzyme-linked immunosorbent assay
EDTA: ethylenediaminetetraacetic acid
EU: ELISA units
FIV: feline immunodeficiency virus
FeL: feline leishmaniosis
FeLV: feline leukemia virus
HE: hematoxylin and eosin
HRM: high resolution melting
IHC: immunohistochemistry
IFAT: indirect fluorescent antibody test
ITS1: internal transcribed spacer 1
Fe-SODe: iron superoxide dismutase
Kd: kilodalton
MLEE: multilocus enzyme electrophoresis
PBS: phosphate-buffered saline
qPCR: quantitative polymerase chain reaction
RFLP: restriction fragment length polymorphism
RT: room temperature
WB: western blot
